# Timeliness of online COVID-19 reports from official sources

**DOI:** 10.3389/fpubh.2022.1027812

**Published:** 2023-01-24

**Authors:** Laura Espinosa, Olesia Altunina, Marcel Salathé

**Affiliations:** Digital Epidemiology Lab, School of Life Sciences, École Polytechnique Fédérale de Lausanne, Geneva, Switzerland

**Keywords:** COVID-19, social media, epidemic intelligence, Europe, Africa

## Abstract

**Introduction:**

Making epidemiological indicators for COVID-19 publicly available through websites and social media can support public health experts in the near-real-time monitoring of the situation worldwide, and in the establishment of rapid response and public health measures to reduce the consequences of the pandemic. Little is known, however, about the timeliness of such sources. Here, we assess the timeliness of official public COVID-19 sources for the WHO regions of Europe and Africa.

**Methods:**

We monitored official websites and social media accounts for updates and calculated the time difference between daily updates on COVID-19 cases. We covered a time period of 52 days and a geographic range of 62 countries, 28 from the WHO African region and 34 from the WHO European region.

**Results:**

The most prevalent categories were social media updates only (no website reporting) in the WHO African region (32.7% of the 1,092 entries), and updates in both social media and websites in the WHO European region (51.9% of the 884 entries for EU/EEA countries, and 73.3% of the 884 entries for non-EU/EEA countries), showing an overall clear tendency in using social media as an official source to report on COVID-19 indicators. We further show that the time difference for each source group and geographical region were statistically significant in all WHO regions, indicating a tendency to focus on one of the two sources instead of using both as complementary sources.

**Discussion:**

Public health communication via social media platforms has numerous benefits, but it is worthwhile to do it in combination with other, more traditional means of communication, such as websites or offline communication.

## 1. Introduction

Epidemic intelligence is the early identification of potential health threats that must be verified, assessed and investigated to recommend public health measures in order to control them (1). It is based on the data collation and collection (2) from two complementary categories of sources: indicator-based surveillance (IBS), comprising structured data from traditional surveillance systems (e.g., electronic health records), and event-based surveillance (EBS) which is an organized approach to collect unstructured data or signals from media, social media, official websites and restricted public health platforms (3).

In the past years and with an increased use of technologies to automatise the data collection, processing, and visualization, this distinction of IBS and EBS is starting to disappear. The COVID-19 pandemic is a clear example of the sharing of IBS data in a way that resembles an EBS approach. From the early stages of the pandemic, countries around the world shared their epidemiological information on COVID-19 (4) through public reports, dashboards, datasets or social media posts. During the pandemic and with the addition of new relevant epidemiological information, such as vaccination uptake, these reports have evolved, changing which data is being shared, as well as the platforms and formats used for sharing it.

Likewise, the concept of digital epidemiology has evolved in the past years. In 2018, Salathé broadly defined digital epidemiology as “epidemiology that uses digital data [...] that was generated outside the public health system”. A more recent review from Aiello et al. (5) and systematic scoping review from Shakeri Hossein Abad et al. (6) expand on the uses of digital surveillance within public health, including some examples and potential future applications. However, these publications largely focused on the use of non-official and non-traditional sources (e.g., using search query data as syndromic surveillance system) rather than on the use of official non-traditional sources (e.g., official social media accounts or websites from health authorities to track an epidemic).

Furthermore, social media has become a key tool not only for sharing and disseminating data and research on infectious diseases and/or pandemics such as COVID-19 (7) but also as a tool for science communication (8) and analysis of perceived risk and sentiment during a pandemic expressed by the users of the different social media platforms (7, 9, 10). Furthermore, data from social media platforms is included in epidemic intelligence systems at national and international level such as Epidemic Intelligence from Open Sources developed by the World Health Organization (WHO) and the Joint Research Center of the European Commission (11), the R package and Shiny app epitweetr developed by the European Center for Disease Prevention and Control (12), and EpiWATCH managed by the Australian National Health and Medical Research Council (13), among others.

Making epidemiological indicators for COVID-19 publicly available can support public health experts in the near-real-time monitoring of the situation worldwide, and in the establishment of rapid response and public health measures to reduce the consequences of the pandemic. Moreover, making this information publicly available on a timely and consistent basis can impact the effective public engagement during an emergency or pandemic (14–16) which is a key aspect to ensure compliance of response/preventive measures needed to tackle the emergency or pandemic (17–19) and reduce misinformation/disinformation that can jeopardize the public health communication strategies.

The objective of this study is to describe the public health communication strategies on COVID-19 in the WHO regions of Europe and Africa by assessing the timeliness of public official sources, in particular comparing social media platforms and other public sources such as websites or dashboards.

## 2. Materials and methods

### 2.1. Selection criteria for countries and sources, and study period

We have focused this study on the countries and territories (hereby referred to as countries) from the World Health Organization (WHO) African and European regions (4), including 100 countries in total, 47 from WHO African region and 53 from WHO European region.

Selection criteria were established to determine the eligibility of these countries for this study, as it follows: countries with official social media accounts (i.e., from ministries of health or public health institutes) in which updates on COVID-19 cases were available on a regular basis.

For each eligible country, two sources were used: an official social media account and an official website. When several official sources with information on COVID-19 cases were found for the same country, the social media account with more frequent updates and the website from which data could be scraped in an easier manner were used. In addition, WHO updates (20) were used for countries in which no website was found that reported information on COVID-19 cases or for which the developed scraper was not able to extract the date and time of the updates. Since these sources are considered official by default, no validation of the data and its credibility were deemed necessary.

The selection of the type of social media platform or type of website was directly related to the preference from the different countries; i.e., official websites were searched for each country that would lead to social media official accounts used and/or most common social media platforms in these two WHO regions were searched (Twitter, Facebook and Telegram) for the official accounts in case the social media official accounts were not indicated in the official websites.

We initially developed a pilot for countries in the WHO European region and, when the methodologies for the social media platforms and websites were stable, we considered it as the started period for this region. In addition, in order to also include another WHO region and more middle- and low-income countries, countries in the WHO African region were included in the study at a later stage. So, the study period was from 31 January to 23 March 2022 for countries in the WHO European region and from 13 February to 23 March 2022 for countries in the WHO African region.

### 2.2. Manual extraction of date and time for the updates on COVID-19 cases in social media platforms

The date and time at which information on COVID-19 cases was updated in social media platforms were manually scrapped with the following metadata: date, time (Central European Time, CET), country, WHO region and source.

The earliest time for each date was recorded in case of several daily updates. In addition, if the update was posted the day after before 5:00 CET, the update was saved on the corresponding reporting day with the time as 23:55 CET.

### 2.3. Automated extraction of date and time for the updates on COVID-19 cases on official websites

We set up automated detection of the websites' update dates and times. For this, we developed a Python-based web scraper. To download the data, we used the Unix wget command and Selenium's automated browsers. To parse the data, we used Beautiful Soup and Pandas libraries.

First, we picked an appropriate download mode for each country. Simple pages were downloaded by the wget command, while the ones with partial data loading were downloaded by a Selenium browser, which allowed us to wait until specific content was loaded onto the page.

We further created a web scraping pipeline which continuously downloaded the data (approximately once a minute) from the relevant website pages into local files. These files were parsed, compared with the previous version based on the relevant information (COVID-19 total cases for this study), and if this had changed, we kept the updated versions with the date and time of collection.

Finally, we filled out the individual data parsers for each source that would select the relevant information needed for comparison.

We launched the web scraper for the countries from the WHO European region on January 31st, 2022, and then expanded the data parsers to the countries from the WHO African region on March 13th, 2022.

### 2.4. Calculation of timeliness of social media platforms for the updates on COVID-19 cases

R version 4.0.5 and RStudio version 1.4.1103 were used for the calculations and methods explained in this section. The following R packages were used on top of base R, in alphabetical order: flextable, ggpubr, hms, janitor, pacman, rjson, rstatix, tidyverse, webshot2.

The countries were grouped in three geographical regions (hereby referred to as regions): WHO African region, EU/EEA from WHO European region, and non-EU/EEA from WHO European region.

The difference in dates and times between website and social media was calculated as follows:

difference (min) = date/time website – date/time social media.

Negative differences represented an earlier daily update on the website, while positive differences represented an earlier daily update on the social media platforms.

When more than one update in the same day was published in an official website, the earliest update was taken for calculating the difference with the social media platform. Despite the data collection from social media platforms recorded manually only the earliest entry of each date, this same filter was applied as validation and to reduce any error.

The non-parametric Wilcoxon test, which does not require any assumption about the distribution of the data, was applied with a 95% confidence interval to determine whether the time difference for each of the three regions was significantly different depending on which of the two sources (social media or website) had the earliest daily update on COVID-19 cases. The two groups compared were: (a) dates and countries with social media platform as earliest update and its difference with the website update and (b) dates and countries with website as earliest update and its difference with the social media platform update. This comparison was done separately for each WHO region. The effect size (r) was calculated as the z value divided by the square root of the sample size. For each of the three regions, the same test and null/alternative hypothesis were applied as follows: the null hypothesis was that the means and standard deviations of time difference in minutes for each source group (websites and social media platform) was equal and the alternative hypothesis was that these were different.

### 2.5. Code and data sharing

Data and code used for the automated extraction of date and time for the updates on COVID-19 cases on official websites and for the calculation of timeliness of social media platforms for the updates on COVID-19 cases can be found in the Covid Sources Timeliness repository (https://github.com/digitalepidemiologylab/covid-sources-timeliness).

## 3. Results

A total of 62 countries were included in the study: 28 from the WHO African region (Algeria, Angola, Botswana, Burkina Faso, Cape Verde, Chad, Comoros, Côte d'Ivoire, Equatorial Guinea, Eswatini, Ethiopia, Gabon, Gambia, Kenya, Madagascar, Malawi, Mali, Mauritius, Namibia, Niger, Nigeria, Senegal, Seychelles, Sierra Leone, South Africa, Uganda, Zambia and Zimbabwe) and 34 from the WHO European region (Andorra, Armenia, Austria, Azerbaijan, Belarus, Bulgaria, Estonia, France, Germany, Gibraltar, Greece, Israel, Italy, Kosovo, Kyrgyzstan, Latvia, Lithuania, Luxembourg, Malta, Monaco, Montenegro, Netherlands, North Macedonia, Poland, Portugal, Romania, Russia, San Marino, Slovakia, Spain, Switzerland, Ukraine, United Kingdom, Uzbekistan).

There were a total of 2,860 entries from 52 days and 62 countries (Table 1). The most prevalent categories were social media updates only in WHO African region (32.7% of the 1,092 entries), and updates in social media and websites in WHO European region (51.9% of the 884 entries for EU/EEA countries, and 73.3% of the 884 entries for non-EU/EEA countries). The least prevalent category in the WHO African region and in the non-EU/EEA countries of WHO European region was having updates in their website only, with 6.8 and 4%, respectively. The least prevalent category in the EU/EEA countries of the WHO European region was having updates in their social media platforms only.

**Table 1:** Number of dates and countries per type of update and WHO region.

**Region**	**Update**	**Number of entries**	**Percentage**
Africa	1. No update	348	31.9%
Africa	2. Website update	74	6.8%
Africa	3. Social media update	357	32.7%
Africa	4. Social media and website update	313	28.7%
Africa	Overall	1,092	100%
Europe/EU-EEA	1. No update	83	9.4%
Europe/EU-EEA	2. Website update	327	37%
Europe/EU-EEA	3. Social media update	15	1.7%
Europe/EU-EEA	4. Social media and website update	459	51.9%
Europe/EU-EEA	Overall	884	100%
Europe/Non-EU-EEA	1. No update	126	14.3%
Europe/Non-EU-EEA	2. Website update	35	4%
Europe/Non-EU-EEA	3. Social media update	75	8.5%
Europe/Non-EU-EEA	4. Social media and website update	648	73.3%
Europe/Non-EU-EEA	Overall	884	100%

The social media platforms used by public health authorities to report COVID-19 cases were Twitter and Facebook for all regions included in this study and Telegram for the non-EU/EEA countries of the WHO European region.

**Figure 1:**
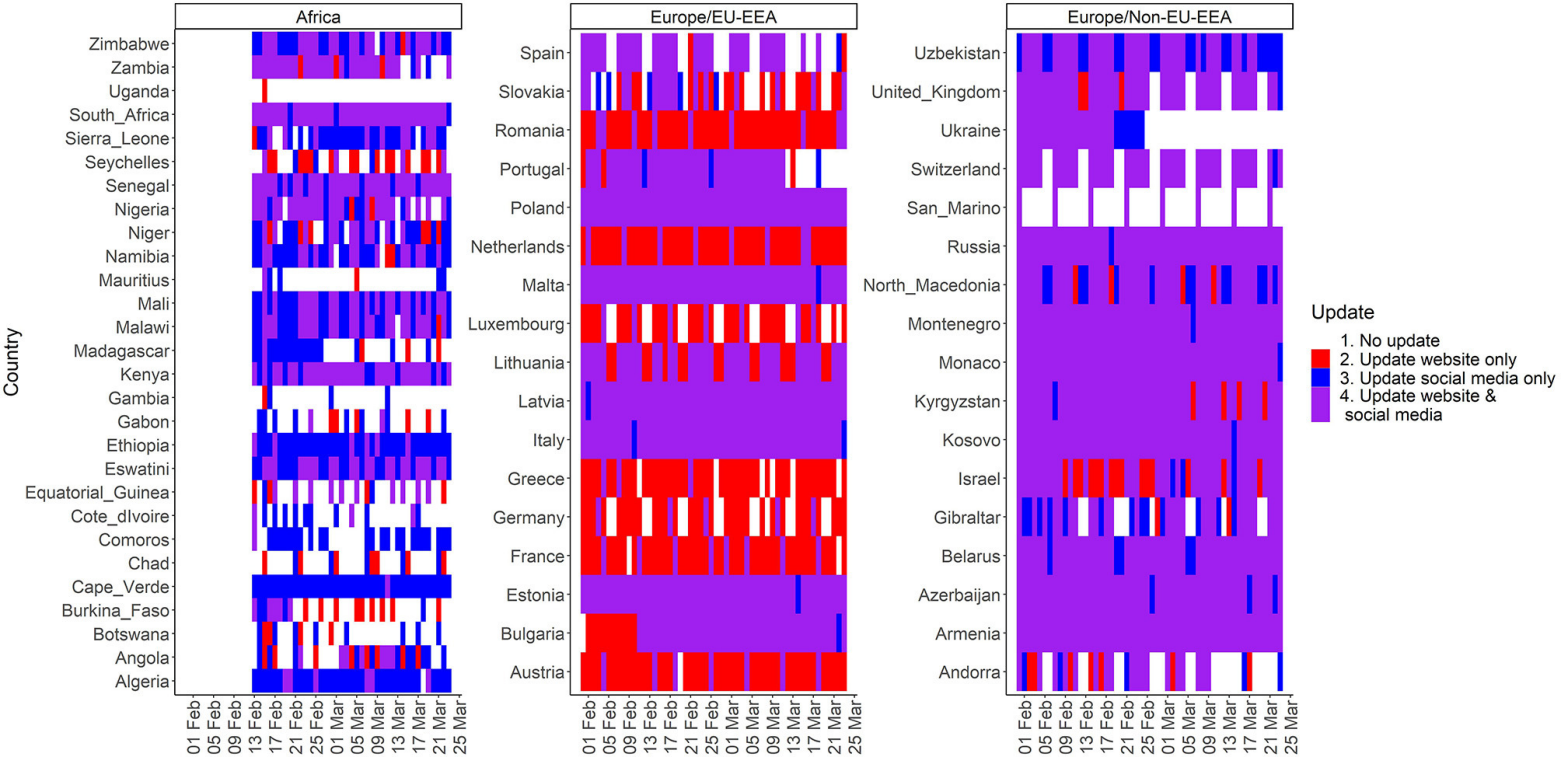
Dates with updated information on COVID-19 cases in websites and/or social media platforms by country and WHO region.

Figure 1 shows the dates for which there was updated information on COVID-19 cases in the websites and/or social media platforms by country and WHO region with four categories: no updates, updates of website only or social media only, and updates of both website and social media. Most of the countries in the WHO European region had a daily reporting of data on COVID-19 cases during the study period with few exceptions in which data was not reported during the weekend (e.g., Spain and Luxembourg) or data was reported on weekly basis (e.g., San Marino). However, in the WHO African region, a minority of the countries reported data on COVID-19 cases on a daily basis (e.g., Ethiopia and South Africa) with greater inconsistencies among the countries.

Table 2 presents the number of entries (i.e., countries and dates) for which updates were available both *via* websites and social media. Social media was the earliest source overall in the WHO European region, whereas websites were the earliest source overall in the WHO African region. There were seven countries in which all updates were earlier on social media (Bulgaria, France, Latvia, Russia, Seychelles, Uzbekistan and Zambia), and nine countries in which all updates were earlier on websites (Austria, Cape Verde, Comoros, Germany, Greece, Lithuania, Madagascar, Mauritius and San Marino).

**Table 2:** Number of entries and percentage for which updates were available in websites and social media platforms for the same country and date per region and earliest source.

**Region**	**Earliest source**	**Number of entries**	**Percentage**
Africa	No difference	0	0%
Africa	Social media	153	48.9%
Africa	Website	160	51.1%
Africa	Overall	313	100%
Europe/EU-EEA	No difference	3	0.7%
Europe/EU-EEA	Social media	256	56.8%
Europe/EU-EEA	Website	192	42.6%
Europe/EU-EEA	Overall	451	100%
Europe/Non-EU-EEA	No difference	2	0.3%
Europe/Non-EU-EEA	Social media	383	59.1%
Europe/Non-EU-EEA	Website	263	40.6%
Europe/Non-EU-EEA	Overall	648	100%

**Figure 2:**
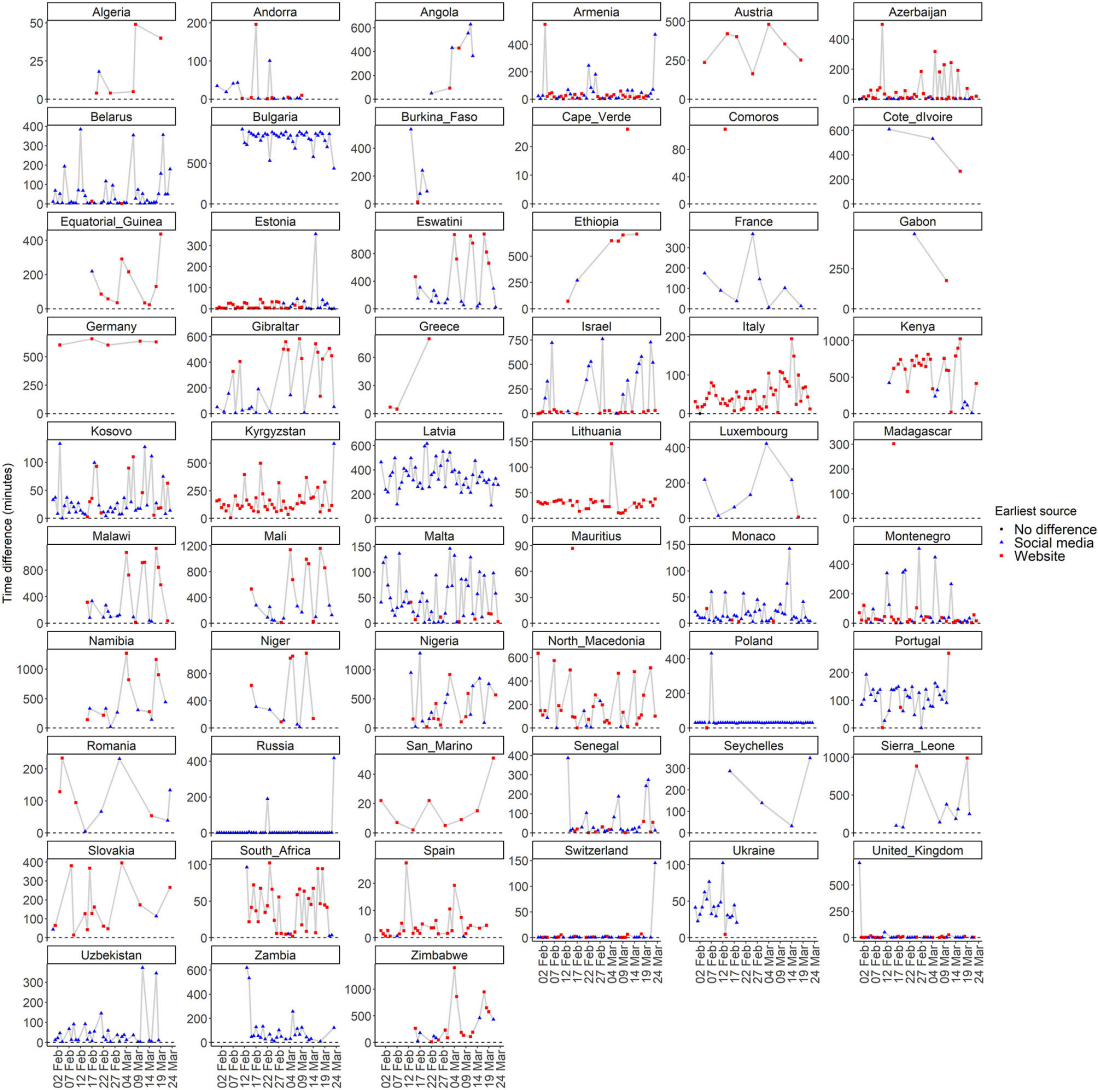
Temporal distribution of absolute time differences in minutes between websites and social media updates on COVID-19 per country.

**Figure 3:**
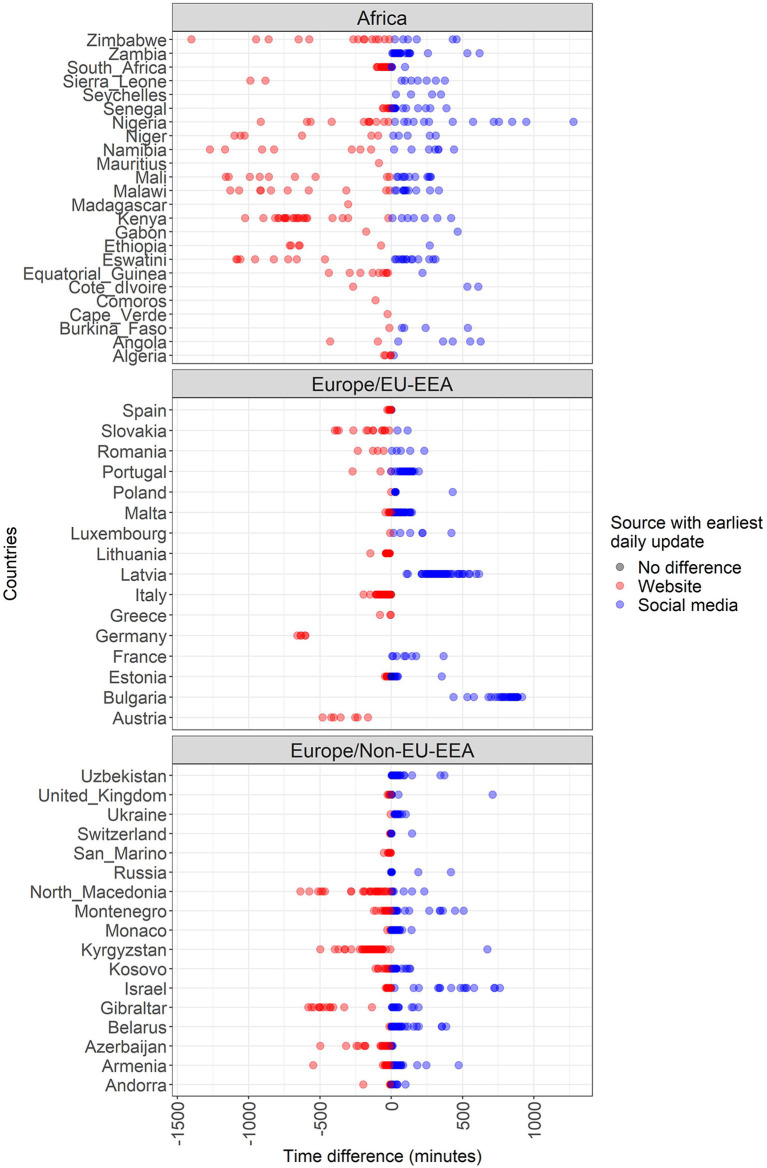
Time differences in minutes between websites and social media updates on COVID-19 cases per country/territory, WHO region and source with the earliest update.

Figures 2, 3 present the time differences in minutes between websites and social media updates on COVID-19 per country. The former focuses on the temporal distribution of the absolute values and the latter focuses on the difference based on the source with the earliest update. Furthermore, Table 3 shows the central measures (median and mean) and measures of dispersion (interquartile range and standard deviation) of the time differences in minutes based on the source with the earliest update.

**Table 3:** Measures of centrality and dispersion of time differences in minutes between websites and social media updates on COVID-19 cases per WHO region and source with the earliest update.

**Region**	**Earliest source**	**Median (min)**	**Interquartile range (Q3-Q1) (min)**	**Mean (min)**	**Standard deviation (min)**
Africa	Social media	108.00	230.55	186.39	206.85
Africa	Website	189.50	670.78	384.49	389.62
Europe/EU-EEA	Social media	105.72	328.67	239.37	281.95
Europe/EU-EEA	Website	26.00	48.36	66.89	124.94
Europe/Non-EU-EEA	Social media	11.20	39.54	57.50	125.70
Europe/Non-EU-EEA	Website	28.88	103.05	92.83	140.58

**Figure 4:**
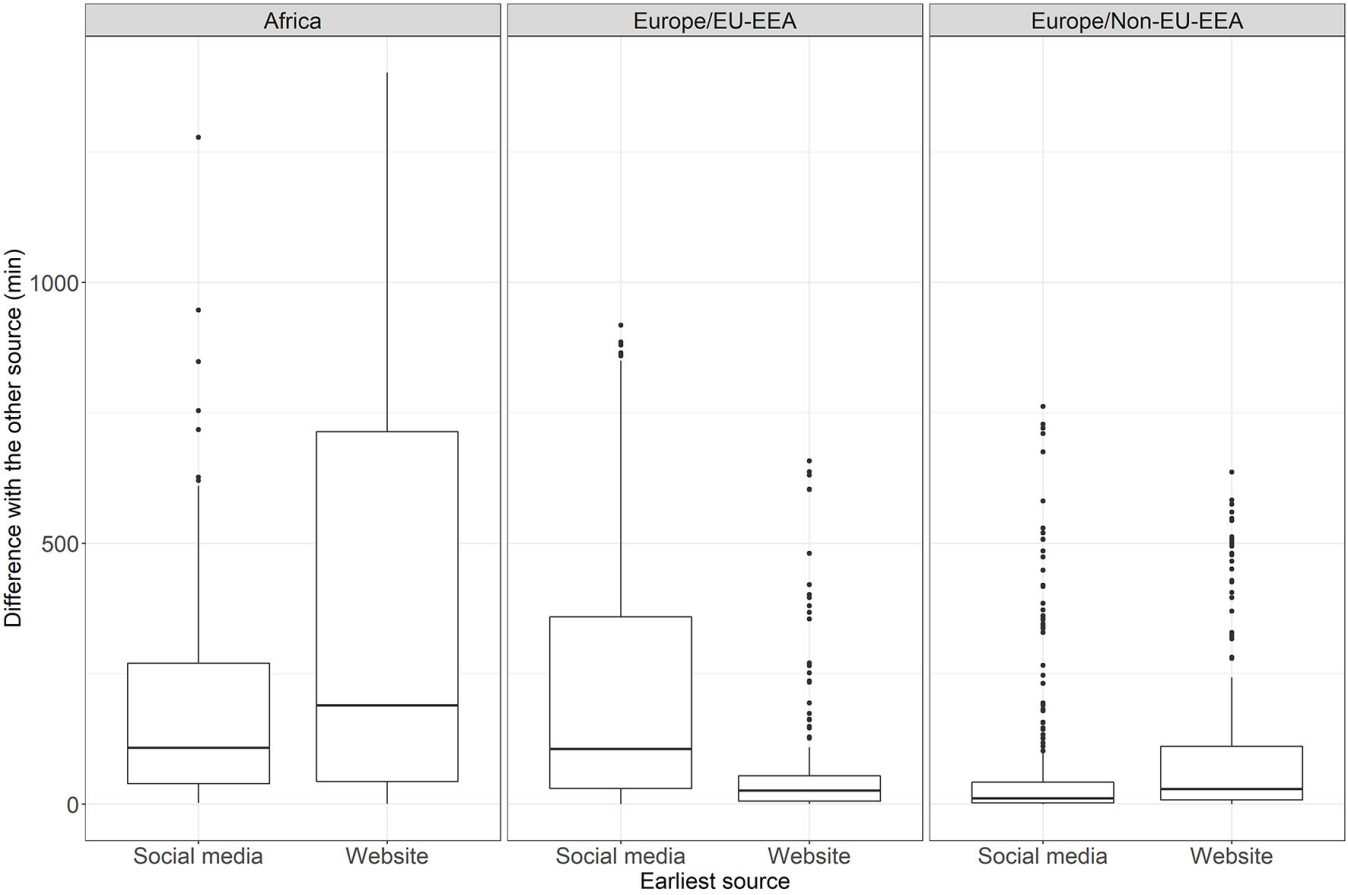
Results of the Wilcoxon test and boxplots on the time difference per source category and geographical region.

The results of the Wilcoxon test on the means and standard deviations of time difference in minutes for each source group and geographical region are shown in Figure 4. These differences were statistically significant in non-EU/EEA and EU/EEA countries and territories from the WHO European region with a small (*r* = 0.243) and moderate (*r* = 0.424) size effect, respectively, and *p-*values lower than 0.0001; and in WHO African region with a small size effect (*r* = 0.170) and *p-*value equal to 0.003.

## 4. Discussion

This study has analyzed the timeliness of two groups of official sources reporting on COVID-19 indicators in the WHO regions of Europe and Africa: websites and social media platforms.

Around 65% of the 2,860 COVD-19 indicator reports in both regions had updates either both in social media and websites, or social media alone, showing a clear tendency in using social media as an official source to report on COVID-19 indicators. In addition, 26 countries from the 62 included in the study (Algeria, Armenia, Azerbaijan, Belarus, Cape Verde, Comoros, Côte d'Ivoire, Estonia, Eswatini, Ethiopia, Italy, Kenya, Kosovo, Latvia, Mali, Malta, Monaco, Montenegro, Poland, Russia, San Marino, Senegal, South Africa, Switzerland, Ukraine and Uzbekistan) had only updates on social media alone, or in social media and websites. This predominance of social media can be due to the ease of posting new information or modifying existing posts as needed in some of the platforms, the possibility of rapidly assessing the impact of this information by the immediate reactions of other users in the platforms, and the increased use by the general population with access to the internet of these platforms as a source of information.

There is higher predominance of the use of social media as a standalone source of information in the WHO African region in comparison to the WHO European region. Already in 2010, it was known that the African region was experiencing a steady increase in the use of internet and social media platforms, especially Facebook, Twitter, and YouTube. In addition, studies showed in the 2000s that most of the time spent online by Africans was on social media platforms with a decrease in the use of the web for reading news (21).

The distribution of the difference between websites and social media platforms per country, region and earliest source showed that most of the countries have different distributions depending on which source was earlier. In addition, the results of the Wilcoxon test on the means and standard deviations of the time difference in minutes for each source group and geographical region were statistically significant in all WHO regions, with a higher confidence in EU/EEA countries of WHO European region (shown by its lower *p-*value and higher size effect). This may indicate that countries tend to focus on one of the two sources instead of using both as complementary sources, which would increase the reach of the information with a positive effect in the general population's awareness of the disease.

Furthermore, there are different reporting strategies (daily, weekly, inconsistent) depending on the country and, more noticeable, depending on the region. These differences may be influenced by the population of the reporting country, indicating a different need depending on the number of cases reported; the availability of resources and data to report more frequently and other factors extrinsic to the pandemic.

The risk communication strategies and effectiveness of public health interventions can benefit from having clear and consistent messages which are directly related to the date and time of publication of the information provided by official authorities. However, the distribution of the absolute difference between websites and social media platforms showed a considerable number of outliers, especially in the non-EU/EEA countries of the WHO European region, indicating a lack of consistency in the publication of the updates which can end up in increased spread of mis-/disinformation.

There are several reasons for which data is not provided in a consistent and timely manner shown by these outliers: data availability, data workflow from local to national level (electronic health records), human power and technologies available to collect, process and publish the information, among others. Having different consistencies in data reporting among the countries and regions included in this study can be explained by the different capabilities and capacities in the aspects mentioned above and show the need of having a targeted assessment to better address the individual needs of each country and region. Standardization of electronic health records among hospitals within the same country and/or region and automatization of data workflows, including automated publication, are some of the solutions that could help in providing more consistent and timely information to the general public and public health authorities.

The main two limitations of this study are the study period and regions included, since the results may vary according to these two aspects. Nonetheless, data collected and analyzed in this study have been sufficient to highlight the characteristics in terms of timeliness, earliest source, and consistency of COVID-19 cases' reporting in the included regions and time period. Furthermore, given that the data was collected in the final phase of the pandemic before most countries lifted most or even all pandemic-related measures, this time window likely represents the current state of the art in the regions covered.

In conclusion, social media is being used for communicating on public health events, but not as a unique type of source. Public health communication *via* social media platforms has numerous benefits, but it is worthwhile to do it in combination with other, more traditional means of communication, such as websites or offline communication (e.g., radio or television). In particular, the closed access to privately owned social media platforms can be problematic from a public health point of view, as public health information should be freely available to everyone at all times. It would furthermore be useful to release public health data in accessible format *via* APIs, so that third party services can easily access the data. The latter would also facilitate the simultaneous update of social media and websites.

## Data availability statement

The datasets presented in this study and code used can be found in online repositories. The repositories can be found below: https://github.com/digitalepidemiologylab/covid-sources-timeliness.

## Author contributions

LE, OA, and MS ideated the study. OA developed the Python-based web scraper for extracting the date and time from official websites and drafted the corresponding section in the materials and methods. MS drafted the abstract. LE mapped the online official sources for COVID-19 indicators, extracted date and time of social media platforms, developed the R scripts for analyzing the results, and drafted the rest of the manuscript. All authors contributed to interpretation of the data and editing of the final manuscript, and have seen and approved the manuscript.
